# Recent Progress in Nanomedicine for Melanoma Theranostics With Emphasis on Combination Therapy

**DOI:** 10.3389/fbioe.2021.661214

**Published:** 2021-03-11

**Authors:** Mengqi Guan, Shoujun Zhu, Shanshan Li

**Affiliations:** ^1^Department of Dermatology and Venerology, The First Hospital of Jilin University, Changchun, China; ^2^Joint Laboratory of Opto-Functional Theranostics in Medicine and Chemistry, The First Hospital of Jilin University, Changchun, China; ^3^State Key Laboratory of Supramolecular Structure and Materials, College of Chemistry, Jilin University, Changchun, China

**Keywords:** Melanoma, nanomedicine, combination therapy, imaging guided surgery, diagnosis

## Abstract

Melanoma is an aggressive type of skin cancer with increasing incidence and high mortality rates worldwide. However, there is still a lack of efficient and resolutive treatment strategies, particularly in clinical settings. Currently, nanomedicine, an emerging area in the medical field, is being widely investigated in small animal models to afford melanoma theranostics. However, several problems, such as tumor heterogeneity, and drug resistance treatment with a single therapy, remain unresolved. Previous reviews have primarily focused on monotherapy for melanoma in the context of nanomedicine. In this review article, we summarize the recent progress in the application of nanomedicine for melanoma treatment, with particular attention to combination therapy based on nanomedicine to achieve optimized therapeutic output for melanoma treatment. In addition, we also highlight the fluorescence-guided strategies for intraoperative melanoma detection, especially in the near-infrared imaging window with greatly improved imaging contrast and penetration depth.

## Introduction

Melanoma, resulting from the gene mutations of melanocytes, is the most aggressive skin cancer (Schadendorf et al., 2015). The pathogenesis of melanoma is complex and involves genetic and environmental factors. The increasing incidence of melanoma in developed countries is mostly due to overexposure to sun (ultraviolet light), which is the main risk factor for cutaneous melanoma (Lawrence et al., 2013). The incidence of melanoma is rapidly increasing; in fact, the number of new cases in the Caucasian population has quadrupled in the past three decades, increasing at the annual rate of 1.5% over 10 years in the United States (US) alone (Cancer Stat Facts: Melanoma of the Skin, 2019; Jones et al., 2019). Due to the high mortality rate and poor prognosis, early diagnosis and effective therapies for melanoma are urgently required.

Most melanoma cases develop in the skin rather than in the viscera or mucosa. For early stage or primary cutaneous melanoma, surgical resection is the gold standard of therapy. However, when melanoma progresses to be aggressive or metastasized form, it needs to be treated by certain standard therapies in terms of chemotherapy, radiotherapy, immunotherapy, and/or targeted therapy. For instance, chemotherapy with dacarbazine (DTIC) is the standard treatment approved by the United States Food and Drug Administration (FDA) for metastatic melanoma, possessing a response rate of monotherapy for 10 to 20% and 2–6% for the 5-year survival rate (Kim et al., 2010b; Miller et al., 2016). More recently, adjuvant immunotherapy agents targeting cytotoxic T-lymphocyte-associated protein-4 (CTLA-4) and programmed death-1 (PD-1) regulatory pathway have been used successfully as adjuvant immunotherapy agents for metastatic melanoma. However, of those who received ipilimumab therapy, a monoclonal antibody against CTLA-4, 10 to 15% of melanoma patients experience potentially fatal autoimmune-related side effects (Hodi et al., 2010). Furthermore, a cascade of drawbacks, such as high costs of treatment and immune escape of tumor cells, hamper the popularization of immunotherapy among patients. In 2011, FDA approved a selective oral *BRAF*-mutant inhibitor, named vemurafenib. It is an effective targeted therapy and commonly used for treating unresectable or metastatic melanomas with *BRAF*^*V600E*^ mutations, demonstrating a response rate of 50%, a median duration 6.7 months, and median overall survival 15.9 months. Unfortunately, it is reported that those who received *BRAF* inhibitors therapy were at increased risk for development of squamous cell skin cancers. Moreover, most patients developed drug resistance; finally, half of the patients treated with *BRAF*-targeted therapies relapsed within 6 months (Chapman et al., 2011; Hauschild et al., 2012; Sosman et al., 2012). Thus, because of the heterogeneity in tumor, the efficacy of single therapy shows much limited therapeutic effects. Accordingly, to achieve higher treatment efficiency and overcome these resistance mechanisms, multimodal therapies targeting distinct pathways have been proposed. In addition, it’s critical to establish an optimal dose that retains the therapeutic benefit while balancing adverse events due to drug reactions. Furthermore, successful treatment will also depend on efficient delivery systems that direct delivery of chemical agents and other therapeutic materials to tumor sites.

Nanotechnology creates and uses materials, devices, and systems at the nanometer scale (Jain, 2005). Nanoparticle structures have been widely used as vehicles to deliver imaging labels or cytostatic/cytotoxic drugs to the sites of growing tumors, holding considerable promise for cancer treatment strategies. Nanosized drug delivery systems (NDDSs) contributed to a remarkably extended circulation time of the drug as a result of the protection of preferential accumulation in tumors from enzymatic degradation (Prabhakar et al., 2013). Additionally, NDDSs can afford rational release of therapeutic drugs by enabling targeted delivery. NDDSs also improve the therapeutic index of chemotherapeutic drugs, thereby minimizing the severe systemic toxicity caused by off-target exposure (Ernsting et al., 2012; Ma et al., 2016). Recently, a growing number of studies have shown that NDDSs have become a powerful tool for combining cancer immunotherapy with other therapies (Mottas et al., 2019; Zhou et al., 2020). For example, the use of immunologically modified nanoplatforms to synergize photothermal therapy (PTT) and immunotherapy for the treatment of breast cancer (Zhou et al., 2020). The nanomaterial, black phosphorus, serves as a near-infrared (NIR)-responsive nano-agent to trigger PTT that enables the optimal immunogenic cell death for anti-tumor immunity (Zhao et al., 2020).

Currently, there are few systematic review articles that have focused on nanomedicine for the combination treatment of melanoma. Therefore, in the present review, we will present the current progress in the application of nanomedicine in the monotherapy and combination therapies based on nanomedicine of melanoma. We also discuss the latest advances in imaging detection of melanoma, with a particular focus on NIR imaging-guided diagnosis and surgery for melanoma.

## Diagnosis and Surgical Removal of Melanoma

### Diagnosis of Melanoma

**FIGURE 1:**
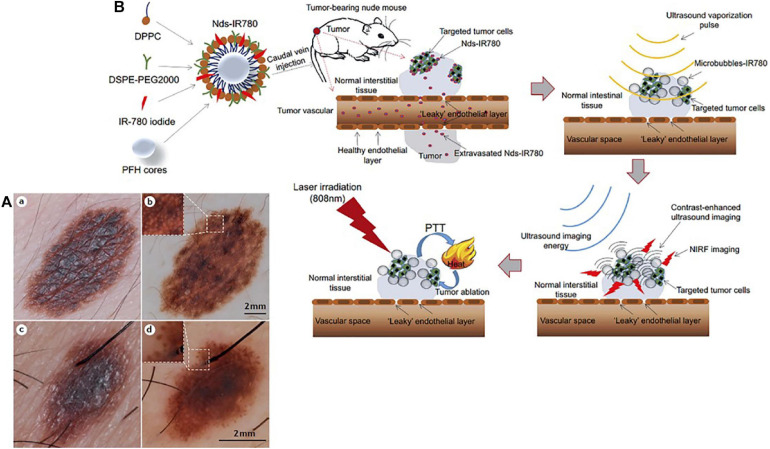
**(A)** Dermoscopy is a non-invasive complementary examination that could examine the detail of minor structures of melanocytic skin lesions. (a) Clinical picture of a several millimeters-scale skin damage with abnormal border and color irregularity. (b) Dermoscopy revealed the appearance with a diagnose of melanocytic naevus. The inset highlights the meshwork pattern. (c) Digital picture of a several millimeters-scale skin lesion. (d) Dermoscopic evaluation revealed a melanoma-specific criterion presented pseudopods at the periphery. The inset highlights the pseudopods. Reproduced with permission (Schadendorf et al., 2015). Copyright 2015, Nature Publishing Group. **(B)** Nds-IR780 preparation. Based on the dual-mode probe with both fluorescence imaging and photothermal ability, cutaneous malignant melanoma was successfully ablated. Reproduced with permission (Yang et al., 2019). Copyright 2019, Dove Medical Press Ltd.

**FIGURE 2:**
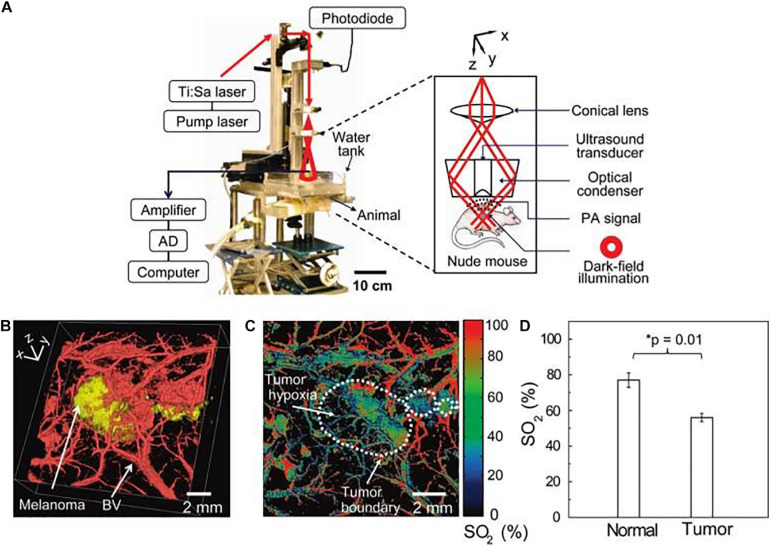
The scheme of home-built photoacoustic (PA) set-up enables high-resolution B16 melanomas intravital PA imaging through a noninvasive manner. **(A)** The home-built PA set-up affords both dark-field and confocal imaging modes with a high level of variability. **(B)** Three dimensional two-color PA imaging of B16 tumor site with both tumor signal (yellow) and vessels shapes (red). **(C,D)** Two-color PA imaging with 564 and 570 nm wavelengths can also provide tumor hypoxia imaging and O_2_ saturation (SO_2_) estimates. Reproduced with permission (Kim et al., 2010a). Copyright 2010, American Chemical Society.

Advanced melanoma is hard to cure and causes high mortality in clinical setting due to a high rate of metastasis and invasion. Therefore, early diagnosis is extremely critical to achieve high survival rates and prolong overall survival in melanoma patients. Generally, most melanomas are detected and diagnosed through a clinical history and systemic examinations including both blood tests and imaging inspection. Skin examination tends to be performed by an experienced physician due to the complexity of pigmentation and morphological patterns. Histopathological examination remains the diagnostic gold standard for melanoma cases that are difficult to diagnose in the clinic. However, biopsy-based methods are invasive procedures with inherent limitations. Moreover, a lack of definitive objective, highly repeatable criteria that are suitable for all melanomas and the absence of molecular diagnostics and prognostic stratification factors contribute to the inconsistency in histopathological diagnosis of melanoma, even for experts in the field (Cerroni et al., 2010; Shoo et al., 2010). In recent years, non-invasive imaging techniques, such as dermoscopy and confocal laser microscopy have proven to be of great value in improving the accuracy of diagnosis of melanomas (Figure 1A) (Vestergaard et al., 2008; Schadendorf et al., 2015; Zhang et al., 2018; Agozzino et al., 2019). Notably, nanotechnology based bioimaging has proven to be a beneficial tool for remarkable improvement of the diagnosis of melanoma. Kim et al. demonstrated that the use of gold nanocages (AuNCs) as an excellent contrast agent for photoacoustic tomography (PAT) could be capable of active targeting the diagnosis of melanomas *in vivo*, increasing the sensitivity and specificity. They demonstrated that the melanocyte-stimulating hormone conjugated AuNCs obtained a 300% imaging contrast enhance in melanoma tumor model when compared to that of the control nanocages (Figure 2; Kim et al., 2010a). Yang et al. (2019) revealed a combination of dual-mode molecular-targeted ultrasound and NIR fluorescence imaging by a novel contrast agent named Nds-IR780 to precisely detect the primary lesions and sentinel lymph nodes (SLN) in cutaneous malignant melanoma. Moreover, Nds-IR780 could also serve as a theranostic probe for targeted therapy in cutaneous malignant melanoma (Figure 1B). Since the tissue autofluorescence and scattering faded with further increase of detection wavelength, imaging in the NIR range can significantly improve the imaging quality in terms of imaging contrast and penetration depth (Tao et al., 2013; Hong et al., 2017; Liu et al., 2019; Zhu et al., 2019). The targeted method can further decrease normal tissue exposure and improve signal at the tumor site. Thus, targeted molecular imaging methods are good approaches to improve the accurate rate of cancer cells detecting.

### Surgical Removal for Melanoma

**FIGURE 3:**
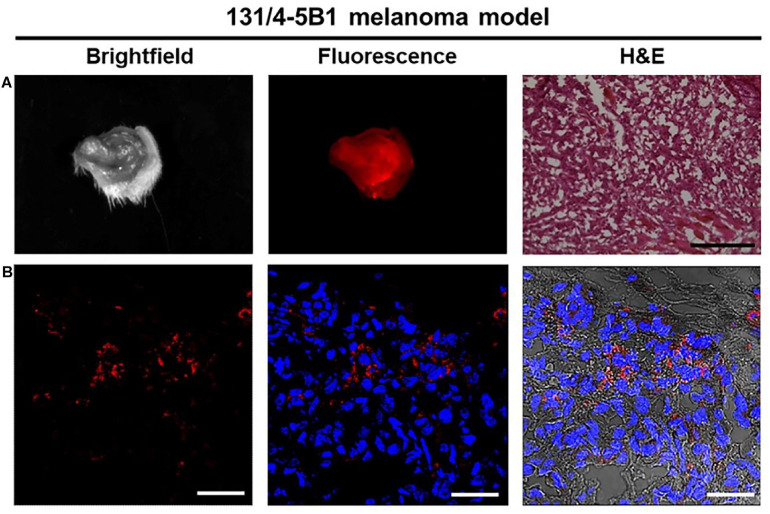
Fluorescence imaging of 131/4-5B1 xenografts injected with (Poly-L-α-glutamic acid)-Cy5-Quencher (PCQ) provides a histology excision with a region of interest (ROI). **(A)** Wide field imaging with both white light and Cy5 fluorescence excitation can confirm the ROI pathological area (10 μm thickness for the tissue section, scale bar 50 μm). **(B)** Red fluorescence imaging indicates the positive tumor signal with scale bar 200 μm. Reproduced with permission (Blau et al., 2018). Copyright 2018, Ivyspring International Publisher.

Surgical resection is the most curative and widely used treatment for cancer patients and is currently considered the optimal choice for majority of patients with resectable solid tumors. Moreover, complete tumor removal during surgery provided the best predictor for patient survival. It has been reported that surgery cured ∼45% of all patients with cancer by removing the whole tumor during the surgical resection (De Grand and Frangioni, 2003). In melanoma, the primary treatment for localized disease (primary tumors) was performed based on the Breslow thickness of the lesions to enable wide local excision with different safety margins (Cancer Genome Atlas Network, 2015). Nevertheless, surgical resection is not the best option for the advanced metastatic melanoma patients, although there are evidences that resection of distant metastases are associated with improved survival rate for the patients with a good state (Gamboa et al., 2020). The identification of surgical margins is a critical surgical task for surgical resection, and the clinicians should ensure a complete resection of the tumor while minimizing damages for surrounding healthy tissue. Thus, it’s necessary and urgent to develop novel technologies and methods that helping in defining the tumor margins, differentiating residual tumor cells, and verifying the complete resection. Blau et al. (2018) developed three polymer-based Turn-ON fluorescent nanoprobes which could be activated by cathepsin enzymatic degradation to generate a fluorescence signal. Their findings showed that the polymeric Turn-ON nanoprobes could delineate tumors boundaries precisely in two orthotopic tumor models of mammary adenocarcinoma and melanoma *in vivo*, largely benefiting the surgical resection (Figure 3). By using quantum dots (QDs) tagged with antibodies, Gao et al. (2004) demonstrated the antibodies labeled QD have the capacity for tumor localization with both targeting and imaging abilities *in vivo*. Apart from the wide local excision of the tumor, careful examination of the regional nodes for metastatic disease spread is also essential for accurate staging melanoma in its early phases. Nanoparticles have been used to detect and visualize SLNs by intraoperative NIR fluorescence imaging. Bradbury et al. (2013) used ^124^I-(cyclic arginine-glycine-aspartic acid-tyrosine)-(methoxyterminated polyethylene glycol)-(core–shell silica nanoparticles) (^124^I-cRGDY-PEG-C dots) coupled with optical imaging devices to localize SLNs in melanoma animal models with promising results. As such, QDs were injected around melanoma tumors in a spontaneous melanoma model in small pigs, and it was found that SLNs imaged in real time were identified in all injections. Furthermore, nodal tissue was resected and histologically confirmed after tissue resection (Tanaka et al., 2006). Collectively, these studies suggest that nanoparticles have multilineage potential in surgical excision.

## Nanomedicine Monotherapies for Melanoma

**FIGURE 4:**
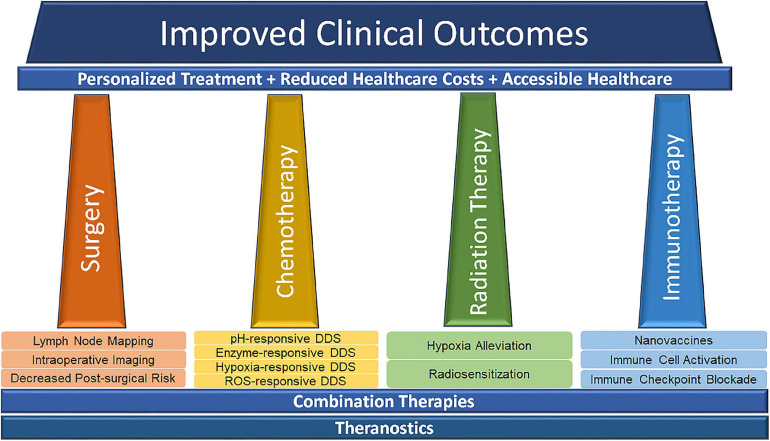
Nanomedicine-related advances in modern cancer treatment. Reproduced with permission (Siamof et al., 2020). Copyright 2020, Frontiers Media S. A.

The implication of nanomedicine in the development of therapies for melanoma is manifold and can significantly improve the prognosis of melanoma patients. The applications of nanotechnology have brought favorable improvements to traditional therapeutic techniques. The final clinical outcomes can be improved through certain therapies and/or combination therapies, including personalized treatment, reduced healthcare, and accessible healthcare in clinic (Siamof et al., 2020; Figure 4).

### Radiotherapy for Melanoma

Radiotherapy is one of the major therapeutic modalities for tumor treatment in the clinical practice. In addition to relying on inducing DNA damage, interrupting the cell cycle, and causing tumor cell death through apoptosis and necrosis, are considered the main mechanisms of radiotherapy with conventionally fractionated radiation. The radiotherapy can also induce neoantigen formation, tumor antigen presentation, and cytokine release to enhance immune responses (Baskar et al., 2012; Takahashi and Nagasawa, 2020). Although radiotherapy often fails to eradicate the tumor completely, it is indeed another adjuvant treatment for the majority melanoma patients who were not suitable for surgical recession, or the advanced nodal disease or primary tumor after definitive resection (Takahashi and Nagasawa, 2020). Adjuvant postoperative radiotherapy may improve local-regional control (LRC) in patients at high-risk of locoregional recurrence after surgery alone; however, overall and disease-free survival rates appear unaffected by this approach (Henderson et al., 2015; Mendenhall et al., 2017). At present, numerous researchers are searching for achieving more efficient and accurate dose delivery to the targeted organs and tissues and thereby reducing the damage to normal tissues and side effects of radiotherapy such as local discomfort.

Currently, the application of high atomic element nanomaterials as radiosensitizers is certainly of significant interest of researchers in radiation oncology and gained widespread attention (Liu et al., 2018). Chang et al. (2008) reported that gold nanoparticles (AuNP) radio-sensitized melanoma cells in a colony formation assay; moreover, AuNP conjugated with ionizing radiation has been found to promote apoptosis of the tumor cells and prolong survival compared to the control group, indicating AuNPs could enhance the effect of radiotherapy. Similarly, Kotb et al. (2016) focused on enhancing radiotherapy utilizing small Gd-based nanoparticles, named AGuIX^®^, and found it was useful as an adjuvant for radiation therapy. They demonstrated that combining AGuIX^®^, with radiation therapy magnified the effect of radiotherapy *in vitro*, thus leading to a decrease in the number of tumor cells and enhancing the survival rate in mice presenting with multiple brain metastases of melanoma. Overall, although nanoparticles have opened up new possibilities for the development of cancer radiotherapy and improvement of clinical efficacy of radiotherapy, many challenges still remain in terms of safety concerns, biocompatibility, and stability.

### Chemotherapy for Melanoma

Chemotherapy, as mono- or poly-therapy, has been the backbone of systemic treatment for melanoma for many years. DTIC remains the only FDA- and EMA-approved chemotherapy drug for treating advanced malignant melanoma and has been used as the standard in most trials of newer agents. It has been reported that the response to DTIC was only 7% in a clinical trial of 64 patients (Johnson et al., 2016). In addition, many other drugs have been used off-label, including temozolomide, fotemustine, paclitaxel, docetaxel, cis/carboplatin, and nitrosoureas. However, single-agent chemotherapy has little role in advanced cutaneous melanoma and is not related to survival benefit, although some response may be observed in certain individual patients. Eigentler et al. (2003) undertook a systematic review of 41 randomized studies and showed that polychemotherapeutic schedules are associated with a higher objective response rate, but is also often more toxic; none was proven to prolong overall survival. This could be attributed to several severe limitations such as systemic toxicity, serious side effects, multi-drug resistance. All these limitations hamper the adequate drug accumulation in the tumor, frequently compromising the efficacy and persistence of the treatment.

The strategy of nanomaterial carrying drugs has been proved to be effective in the field of chemotherapy as they can protect the carried drugs against degradation thus preserving their long-term stability and efficacy. NDDSs can be adjusted to release their cargo only at the tumor site with a response to the specific scenario around the tumors thus reducing cytotoxic side effects on normal tissues (Gmeiner and Ghosh, 2015). Chaudhuri et al. (2010) showed a combination of doxorubicin (DOX) and carbon nanotube in mouse melanoma models could diminish the drug systemic toxicity without sacrificing its therapeutic effect, thus abrogating tumor growth. In addition, Yoncheva et al. (2019) encapsulated DOX into chitosan/alginate nanoparticles and evaluated the therapeutic response *in vitro*/*in vivo* in melanoma cells. The results showed that the encapsulated DOX increased intracellular accumulation of the drug and produced a long-lasting cytotoxic effect thus reducing both the viability of melanoma cells and the tumor size in melanoma mouse models. Similar results have been reported in a study where a trinary tumor accumulation of DOX was seen in animals developing melanoma, using polymeric nanoparticles in comparison to the commercially available PEGylated-liposomal DOX (Zou et al., 2016).

### Immunotherapy for Melanoma

Melanoma has been recognized as an immunogenic cancer eliciting striking antitumor responses for long time. Immunotherapy, mainly including vaccines, bio-chemotherapy, and the transfer of adoptive T cells and dendritic cells, aimed at priming the immune system and activating immune responses to the residual tumor cells (Rodríguez-Cerdeira et al., 2017). Immunotherapy can reduce the size and involvement of locally advanced melanoma, converting inoperable tumors to operable status. Patients with advanced unresectable melanoma may receive high-dose interleukin-2 monotherapy approved by FDA in 2011, meanwhile, the PEGylated interferon-α2b could be used as an adjuvant treatment for surgically treated melanoma patients (Herndon et al., 2012; Tarhini et al., 2012). In addition, significant progress has been made in targeting the immune checkpoints of tumors with PD-1 and CTLA-4. But the efficacy of the checkpoint inhibitors of the immune system was not observed for all patients, and their success rates are often less than 50%, suggesting significant room for improvement (Chen and Mellman, 2017).

A recent study reported a magnetic nanomedicine-conjugated anti-PD-L1 and T-cell activators may enter the tumor site by the magnetic navigation, inhibiting tumor growth *in vivo* and enhancing the tumor microenvironment immunity. This kind of modality may present a novel approach for nanotechnology derived immunotherapy (Chiang et al., 2018). Although this study was not done in melanoma model, it is very promising for future melanoma therapy. Fontana et al. (2019) also revealed that the biohybrid nano-vaccine with a checkpoint inhibitor could enhance the efficacy of the immunotherapy, thereby retarding the tumor growth compared to the standard monotherapy with checkpoint inhibitor.

### Targeted Therapy for Melanoma

Approximately 70% of patients with cutaneous melanoma have gene mutations in the key signaling pathways (Flaherty, 2012). Targeted therapy, a crucial systemic treatment, is a novel method through using small molecule inhibitors or antibodies to affect oncogenic sites. In melanoma, these small molecule inhibitors primarily include *BRAF* inhibitors, MEK inhibitors, CKIT inhibitors, vascular endothelial growth factor inhibitors, P13K-AKT-mTOR pathway inhibitors, cyclin-dependent kinase inhibitors, and receptor ErbB4 inhibitors (Domingues et al., 2018). Approximately 50% of metastatic melanoma lesions have mutations in the serine-threonine kinase *BRAF*, mostly that of valine to glutamine in codon 600 (Davies et al., 2002). At present, the high and rapid response rates as well as good safety profile of *BRAF*-targeted agents offer an unprecedented opportunity for a neoadjuvant approach in melanoma treatment.

Siu et al. (2014) designed non-covalently functionalized nanotubes as a delivery vector for small interfering RNA (siRNA) targeting *BRAF* gene silencing. The uptake of *BRAF* siRNA in melanoma tissue has been observed, resulting in regressing the tumor growth in mouse melanoma models. Similarly, Tran et al. (2008) used a unique nano-liposomal ultrasound to deliver siRNA specifically targeting ^*V600E*^B-Raf and Akt3 into cutaneous melanoma, suppressing the tumor development and preventing the metastasis.

## Nanomedicine-Based Combination Therapy for Melanoma

Nanomedicine with multifunctional design greatly increases the sensitivity of cancerous cells to drugs, reactive oxygen species (ROS), X-ray radiation, and heat, thus improving the therapeutic effects of several treatments in a synergistic manner. In this section, several typical multimodal synergistic therapies are discussed to establish an optimized therapeutic output for melanoma and melanoma-relevant therapy. This section also gives particular focus on combination therapy (imaging-guided surgery plus multimodal synergistic therapies) for melanoma along with the clinical potential of this technique.

### Synergistic Radio/Photothermal/Photodynamic-Therapy

Daneshvar et al. demonstrated the use of platinum nanoparticles (PtNPs) could be served as the absorber for laser light and X-rays to perform energy conversion. Moreover, it could also be a sensitizer that could be applied for the treatment of cancer. Conventional radiotherapy shows low efficacy due to the administration of low radiation doses for safety and the weak absorption of radiation beams by soft tissues tumors. Hence, the authors demonstrated PtNPs provided a deeper treatment and producing more ROS with laser light radiation in the B16/F10 cells, compared to those with sole laser radiation or X-rays (Daneshvar et al., 2020). Following a similar approach, Salehi et al. (2019) utilized platinum mesoporous as a novel NIR photosensitizer for PTT and X-ray absorbing agent for X-ray-induced photodynamic therapy (X-PDT) for melanoma cancer cells. The combined exposure (PTT/X-PDT) led to deeper treatment and very low survival rate of melanoma cells and higher effectiveness and cooperativity compared to that with PTT or X-PDT alone.

### Synergistic Sono/Chemo/Photothermal-Therapy

**FIGURE 5:**
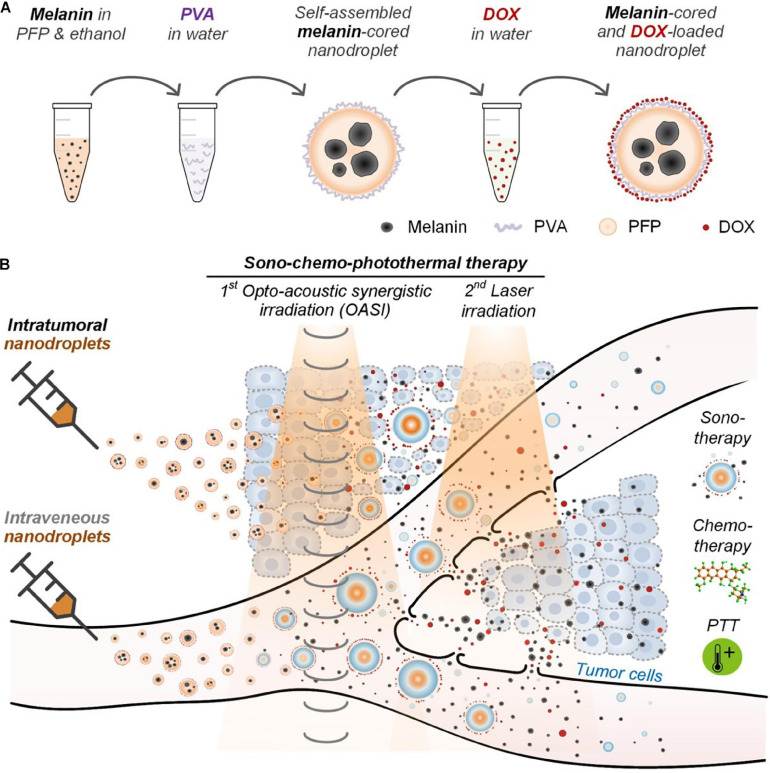
**(A)** Melanin- and DOX-loaded nanodroplet enables **(B)** sono-chemo-photothermal therapy (PTT) trimodal tumor therapy through both intravenous and intratumor injections. Reproduced with permission (Hu et al., 2020). Copyright 2020, Ivyspring International Publisher.

A combination of the mechanical wounding, cytotoxic and photothermal effects associated with, respectively, ultrasound contrast agents, chemotherapy drugs, and melanin were used for a sono-chemo-photothermal cancer therapy. Hu et al. (2020) developed self-assembling perfluoropentane nanodroplets that exhibited excellent biocompatibility for melanoma therapy. Polyvinyl alcohol (PVA)-shelled and melanin−cored nanodroplets were spontaneously generated by using the Ouzo effect, and then anti-cancer drug DOX was loaded into PVA shells of the nanodroplets (Lepeltier et al., 2014). Then, following the loading process, opto-acoustic irradiation was administrated to manufacture the nano-cavity structure through the nanodroplets vaporization. The results showed that the tumor structures were mechanically disrupted in *in vivo* animal experiments, and the nanodroplets, combined with opto-acoustic synergistic irradiation treatment and thereafter by laser irradiation could efficiently eliminate melanoma tumors in both *in vitro* and *in vivo* experiments (Figure 5).

### Synergistic Chemo/Immuno/Photothermal-Therapy

Qin et al. presented a combination therapy, based on a α-cyclodextrin (CD)-based gel system, that combined DOX, with indocyanine green (ICG), a photothermal agent, and CpG, a molecular adjuvant capable of inducing DC mutation. The as-prepared chemo/immuno/photothermal-therapy strategy can effectively inhibit the primary tumor growth. Unexpectedly, the inclusion of PD-L1 blocking obstructed tumor growth in both primary and metastatic melanoma and reduced the tumor size and inhibited the tumor recurrence (Qin et al., 2020). The combinatorial therapy process is very effective due to the sustainable release of tumor-specific antigens. Moreover, compared with other immunotherapy strategies, this novel therapy developed a powerful tumor-specific immunity which will potentially cover a wide range of solid tumors.

### Synergistic Immuno/Photodynamic-Therapy

**FIGURE 6:**
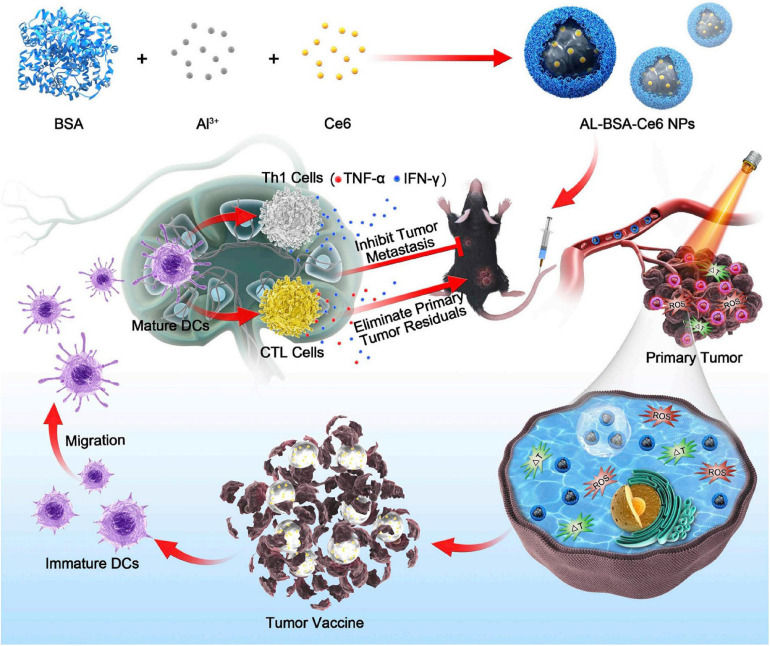
Aluminum ions/Ce6 BSA complex immunotherapy is schematically depicted. Tumor cells are destroyed by photodynamic therapy (PDT) and photothermal therapy (PTT) combination-therapy with aluminum ions/Ce6 BSA complex. The immature dendritic cells (DCs) further gather tumor antigens when they migrate to adjacent lymph node. Then, the proliferation of Th1 cells and cytotoxic lymphocytes (CTLs) could be triggered by mature DCs, eliminating residual tumor cells within the primary tumor and inhibiting metastasis. Reproduced with permission (Zhu et al., 2020). Copyright 2020, Elsevier BV.

Zhu et al. described a novel nano-system [Al-bovine serum albumin (BSA)-Ce6 NPs] that combines immunotherapy with PDT using chlorin e6 as photosensitizer and the aluminum hydroxide as immunoadjuvant. They demonstrated that the nanoparticles not only effectively killed melanoma cells but also prevented tumor relapse or metastasis by inducing potent antitumor immune response, thereby enhancing the immune response indicators levels such as serum antibody, the levels of cytokine and increasing the proportions of immune cells (Figure 6; Zhu et al., 2020). Collectively, melanoma treatments have ushered a new era, as the combination therapy could boost the therapeutic effects with a never-before-achieved effectiveness.

Nanomedicine-based combination therapy for melanoma may reduce high drug resistance, long-term toxicity and increase the efficacy associated with conventional treatments and monotherapies. Therefore, the combination therapy based on nanomedicine could provide the respective advantages of monotherapy and reduce recurrence and metastasis, improving the survival rate to some extent.

## Conclusion and Perspective

Malignant melanoma is associated with the highest risk of mortality among all types of skin cancer, in addition, its incidence is increasing. Due to its rapidly metastasizing and drug-resistance properties, combination treatment has obvious advantages than monotherapy. With the rapid development of nanotechnology, nano-based drug carriers and materials have many advantages including the high sensitivity in detection, reduction in systemic toxicity of chemotherapy drugs and prevention of drug resistance, making them generate a lot of medical benefits in recent years, especially in the field of tumor therapy. Nanomedicine has expanded the scope of diagnosis and therapeutic applications of melanoma, and it reduces not only the physical disease burden but also the economic burden of patients. However, some challenges remain and need to be overcome:

1. Currently, nanomedicine for the treatment of melanoma is mostly investigated in animal models and *in vitro* cellular experiments. Although crucial advances have been demonstrated *in vitro*, the transition to clinical settings remain arduous, because the data from murine models cannot exactly reflect the effects on the human body since most rodent tumors grow much faster than human tumors. The development of human-specific *in vitro* cell culture models is urgently needed.
2. In addition to all these therapeutic advantages, nanoparticles also present some drawbacks, such as the agnostic cytotoxicity and biocompatibility. Additionally, the safety and long-term effects of nanoparticles in melanoma therapy are unknown. For example, the aggregation of large nanoparticles in the endoplasmic reticulum, inducing autoimmunity. Thus, different nanoparticles exhibit distinct collateral effects, and if without a proper engineering or functionalization, they could prove fatal (Jabir et al., 2012).
3. As mentioned above, NIR fluorescence imaging, including both 700–1000 nm NIR-I window and 1000–1700 nm NIR-II region, has several advantages in terms of real-time monitoring, intraoperative navigation ability, and reasonable imaging quality (Zhang et al., 2017; Ding et al., 2018; Fan et al., 2018; Qi et al., 2018; Zhong et al., 2019). It is particularly suitable for cutaneous tumors (e.g., melanoma) with a total depth of less than 1 cm. NIR imaging combined with tumor-targeting fluorophores will enable intraoperative cancer diagnosis. The much-improved imaging contrast will afford clear navigation to distinguish melanoma from adjacent nonmalignant tissue.
4. Most importantly, future direction to melanoma theranostic will first rely on NIR imaging-guided detection and excision with clear tumor margins at three-dimensional angles. The second step will apply synergistic combination therapy based on an efficient nanomedicine strategy. To achieve this two-step theranostic, nanoparticles combined with both targeting imaging and multimodal therapy ability are highly needed in both lab-scale small animal models and clinical settings. In addition, the current instruments for NIR fluorophore detection mainly focus on the NIR-I window, and it is important to further move the NIR imaging-guided surgery to the NIR-II window with much improved imaging contrast and penetration depth (Ding et al., 2019; Zhang et al., 2019; Li et al., 2020; Wang et al., 2020; Xu et al., 2020).

## Author Contributions

MG summarized and wrote the manuscript. SZ and SL commented and edited the submitted version.

## Conflict of Interest

The authors declare that the research was conducted in the absence of any commercial or financial relationships that could be construed as a potential conflict of interest.
